# Determination of the Wound Healing Potentials of Medicinal Plants Historically Used in Ghana

**DOI:** 10.1155/2017/9480791

**Published:** 2017-02-23

**Authors:** Sara H. Freiesleben, Jens Soelberg, Nils T. Nyberg, Anna K. Jäger

**Affiliations:** ^1^Department of Drug Design and Pharmacology, Universitetsparken 2, 2100 Copenhagen, Denmark; ^2^Museum of Natural Medicine, University of Copenhagen, Universitetsparken 2, 2100 Copenhagen, Denmark

## Abstract

The present study was carried out to investigate the wound healing potentials of 17 medicinal plants historically used in Ghana for wound healing. Warm and cold water extracts were prepared from the 17 dried plant species and tested in vitro in the scratch assay with NIH 3T3 fibroblasts from mice. The wound healing scratch assay was used to evaluate the effect of the plants on cell proliferation and/or migration in vitro, as a test for potential wound healing properties. After 21 hours of incubation increased proliferation and/or migration of fibroblasts in the scratch assay was obtained for 5 out of the 17 plant species. HPLC separation of the most active plant extract, which was a warm water extract of* Philenoptera cyanescens*, revealed the wound healing activity to be attributed to rutin and a triglycoside of quercetin. The present study suggests that* Allophylus spicatus*,* Philenoptera cyanescens*,* Melanthera scandens*,* Ocimum gratissimum*, and* Jasminum dichotomum* have wound healing activity in vitro.

## 1. Introduction

Treatment of wounds is a frequent indication recorded in ethnopharmacological studies. Many traditional medicines are used for cleaning or treating wounds, but only a few have been tested pharmacologically for their wound healing potentials.

Early historical descriptions of Ghanaian medicinal plants from 1695–97, 1799–1803, and 1817 among the Fante, Ga, and Ashanti, respectively [1–3], include plants used for wound healing. The term “old leg injury” features prominently in the historical documents. This term is interpreted to refer to chronical wounds especially after a guinea worm infection [4]. Guinea worm disease is caused by the parasitic guinea worm,* Dracunculus medinensis. *After approximately one year of infection the female worm emerges through the skin, often in the legs or feet. The escape of the worm from the body is often accompanied by an ulceration of the area from which the worm has emerged [5]. Since the traditional way to remove the worm by winding it around a small stick can be conducted with only a few centimeters of the worm every day, this process can take a very long time, thereby leaving a serious wound [6]. The guinea worm is nearly extinct [7], so the use of medicinal plants for this special condition is not of much relevance today, but chronical wounds still appear as, for example, chronic venous leg ulcers or diabetic foot ulcers.

An extensive ethnopharmacological study of Ghanaian plants for wound healing recorded 104 plant species as being used for wound healing [8]; however only three species (*Aframomum melegueta, Melanthera scandens, *and* Ocimum gratissimum*) were the same as those recorded in previous centuries. This could be a reflection on the study areas not being at the same geographical and cultural areas of Ghana. None of the three species used historically was included in the subsequent in vitro testing of selected plant species [9].

The wound healing process for acute wounds consists of four phases: hemostasis, inflammation, proliferation and migration of cells, and tissue remodeling or resolution [10].

Hemostasis begins immediately after the injury and involves vascular constriction and aggregation of platelets to form a fibrin clot, from where proinflammatory cytokines and growth factors such as transforming growth factor, epidermal growth factor, fibroblast growth factor, and platelet-derived growth factor (PDGF) are released [10].

When inflammation begins neutrophils clear the area for invading microbes and cellular debris around the wound and macrophages clear the area for apoptotic cells. Macrophages also stimulate other cells to promote tissue regeneration, thereby playing a role in promoting the next stage of wound healing, the proliferation and migration of cells [10].

Both endothelial cells and fibroblasts are present in the reparative dermis of the skin [10]. Fibroblasts and endothelial cells are attracted by mediators produced by inflammatory cells in the wound, and the cells proliferate to expand and migrate into the wounded area [11, 12]. Within the wounded area fibroblasts produce various compounds including collagen, which is a major component of the skin extracellular matrix [10, 11]. Skin fibroblasts can change their character; for example, in a wound they can change their actin gene expression and take on some contractile properties of smooth muscle cells and in this way help to pull together the etches of the wound. Such fibroblasts are called myofibroblasts [11].

Fibroblasts can be arrested in a specialized nondividing state called the G_0_ phase until they are triggered to proliferate by a growth factor or other extracellular signals. There are many proteins that act as mitogens, but PDGF is believed to have an important role in stimulating cell division during wound healing [11].

The fourth and last phase of wound healing is scar formation and remodeling of the tissue. An important part of this phase is the extracellular matrix attaining the architecture of normal tissue. Therefore, fibroblasts also have a role in this phase of wound healing [10].

A scratch assay has been used as an in vitro model of wound healing in a few studies of medicinal plants [13–18]. In the present study fibroblasts are used to resemble the third phase of wound healing, proliferation and migration of cells into the wounded area. A monolayer of cells is grown in medium supplemented with serum and the cell layer is scratched with a pipette tip to imitate a wound. Plant extracts are then tested in the assay to see if they increase the proliferation and/or migration of the cells.

The present study aims to investigate the wound healing potentials of plants historically used for this purpose, as a part of a larger research collaboration, which aims to examine historical and contemporary medicinal plants in Ghana [4].

## 2. Materials and Methods

### 2.1. Cell Line and Chemicals

NIH 3T3 fibroblasts were purchased from Institute of Pharmacy, University of Copenhagen. The cells were maintained in Dulbecco's modified Eagle's medium supplemented with 10% Fetal bovine serum and kept at 37°C with a CO_2_ supply of 5%.

PDGF was purchased from Invitrogen Gibco. Rutin and Sil-A were purchased from Sigma.

### 2.2. Plant Material

Seventeen plants traditionally used for wound healing in Ghana were collected in Ghana from November 2013 to January 2014. Plant material was air-dried away from sunlight and stored in airtight bags. Voucher specimens were identified by Jens Soelberg and deposited at the Herbarium of University of Copenhagen (C) and Herbarium of University of Ghana (GC). Voucher numbers are given in Table 1.

### 2.3. Wound Healing Scratch Assay

Migration of NIH 3T3 fibroblasts was assessed using the wound healing scratch assay. The cells were seeded in 24-well tissue culture dishes for 24 hours at 37°C, at a concentration of 7.6 × 10^4^ cells/mL, and cultured in 1 mL medium containing 10% fetal bovine serum to a nearly confluent cell monolayer.

A linear scratch was created in the monolayer with a sterile pipette tip (Fastrak, 1250 *μ*L Macro Tip, FR1250, Alpha Laboratories Ltd.), and the medium was replaced by 500 *μ*L new medium (control group), 20 ng/mL platelet-derived growth factor, PDGF (positive control), and the crude extracts (10 *μ*g/mL). The experiments were made in triplicate. The cells were incubated at 37°C for 21 hours. Three images were photographed of each well under a Leica DMLS microscope at 4x/0.10 magnification before and after incubation to estimate the proliferation and/or migration of cells. The data were analyzed using Leica application suite, LAS. Cell proliferation/migration rate was calculated as percent closure of the scratch within 21 hours:

(1) Cell  migrationproliferation=gap  distancet0−gap  distancet21gap  distancet0·100%.

### 2.4. Reversed-Phase High-Performance Liquid Chromatography

Reversed-phased HPLC was used to separate the active extracts. A Shimadzu apparatus (LC-20AB, Prominence liquid chromatograph and SPD-M20A, Prominence diode array detector) was used for analytical as well as preparative HPLC. For analytical HPLC the column was a Thermo Scientific C-18 column, 150 × 4 mm, particle size 5 *μ*m, flow: 1 mL/min. For preparative HPLC the column was a Supelco C-18 column, 250 × 10 mm, particle size 5 *μ*m, flow: 5 mL/min.

Mobile phase A was methanol : water (95 : 5) supplemented with 0.1% formic acid. Mobile phase B was methanol : water (5 : 95) supplemented with 0.1% formic acid.

Fractions of* P. cyanescens* were collected while using a linear gradient from 10% B to 80% B from 0–30 min. The chromatograms were recorded at 254 nm, 270 nm, and 310 nm.

### 2.5. NMR

The structural identification of wound healing active compounds was performed using NMR spectroscopy. The fractions were dissolved in 50 *μ*L deuterated methanol and 30 *μ*L was transferred to a 1.7 mm NMR tube. ^1^H-NMR and HSQC spectra were acquired using a 600 MHz Bruker Avance III equipped with a cryogenically cooled 1.7 mm TCI probe head. All spectra were analyzed with ACD/NMR version 12.0 software.

### 2.6. GC-MS Sugar Analysis

Approximately 0.2 mg of fraction 3 was incubated with 200 *μ*L 2 M TFA at 120°C for two hours. The sample was evaporated to dryness with nitrogen and 200 *μ*L of 25 mg/mL hydroxylamine hydrochloride in pyridine was added. After incubation on a water bath at 40°C for 20 min, 100 *μ*L of the resulting sugar-oxime solutions was transferred to an Eppendorf tube and the pyridine was evaporated with nitrogen. Fifty *μ*L Sil-A was added to the tube and it was left for 15 min at 20°C. The resulting solutions were centrifuged in 2 min and 28 *μ*L of the supernatant was diluted with pyridine to 200 *μ*L. Sugar standards of D-glucose, *α*-L(+)-rhamnose monohydrate were prepared in the same way, but with 35 *μ*L of the supernatant diluted with pyridine to 500 *μ*L.

GC-MS spectra were recorded on an Agilent GC-MS system comprising 5973N Mass Selective Detector, 6890N Network GC-system, 7683 Series Injector, and Autosampler (Agilent Technologies, Santa Clara, USA). The system was operated in EI mode at −69.9 eV, recording masses in the range 35.00–400.00. Sugar standards were injected in 1 *μ*L volumes, fraction 3 in 3 *μ*L volume, on an Agilent 19091S HP-5MS capillary column (5%-phenyl-methylpolysiloxane; 30 m × 250 *μ*m × 0.25 *μ*m). The carrier gas (helium) was set to a flow rate of 1 mL/min and the split ratio was set to 1 : 20. The temperature program comprised 125°C for 3 min followed by 125–270°C at 4°C/min. The injector temperature was held at 250°C.

### 2.7. Statistical Analysis

Statistical analyses were performed using Microsoft Office Excel 2010 data analysis. Data are expressed as the mean ± SEM. Significant differences between the test solutions and the control group were determined by a *t*-test or a single-factor ANOVA in Microsoft Office Excel 2010 data analysis with the significance factor *p* < 0.05.

## 3. Results and Discussion

### 3.1. Wound Healing Activity of Plant Extracts

**Figure 1:**
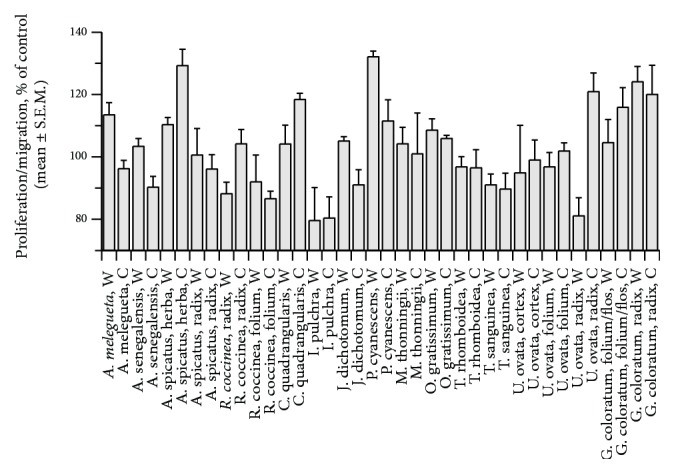
Effect of cold (C) and warm (W) water extracts of plants from Ghana tested for wound healing activity in the scratch assay.

Warm and cold water extracts were tested for wound healing activity in the scratch assay. Extracts of five of the 17 plant species tested showed increased proliferation and/or migration of fibroblasts in the scratch assay (Figure 1). The mean proliferation/migration rate of* Allophylus spicatus* (warm and cold extracts of herba),* Philenoptera cyanescens* (warm extract),* Melanthera scandens* (warm extract),* Ocimum gratissimum* (cold extract), and* Jasminum dichotomum* (warm extract) was significantly higher than that of the negative control group.

A minimum of 120% proliferation/migration rate compared to the negative control group was used as inclusion criteria for further analysis. Therefore, the warm water extract of folium/fructus of* P. cyanescens* was chosen for further analysis.

### 3.2. Isolation of Active Compounds

**Figure 2:**
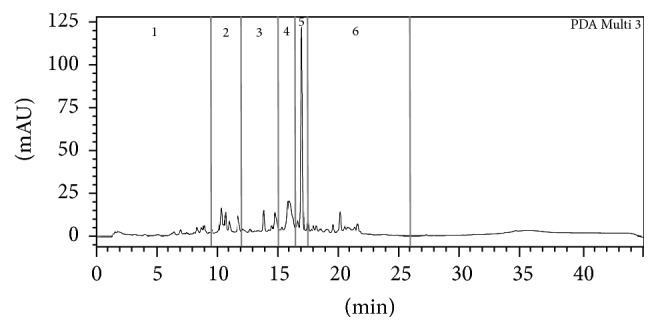
HPLC chromatogram of* Philenoptera cyanescens* at 310 nm separated into fractions 1–6.

**Figure 3:**
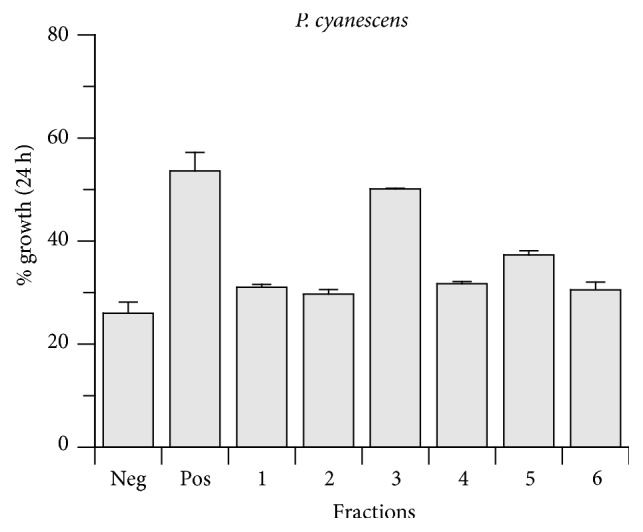
Activity of fractions 1–6 of* Philenoptera cyanescens* tested in the wound healing scratch assay. Neg: negative control, Pos: positive control (20 ng/mL PDGF).

The warm water extract of folium/fructus of* P. cyanescens* was separated in 6 fractions by analytical HPLC (Figure 2). The fractions were tested in the wound healing scratch assay in concentrations corresponding to 10 *μ*g/mL crude extract (Figure 3).

Fractions 3 and 5 of* P. cyanescens* showed significantly more growth than the control group, with fraction 3 showing significantly more growth than fraction 5. The HPLC chromatogram contained two minor peaks in fraction 3, whereas fraction 5 contained the major peak of the HPLC chromatogram. These peaks were isolated by preparative HPLC and tested in the scratch assay, leading to two active compounds, one (**1**) from fraction 3 and the main compound (**2**) from fraction 5.

Compound** 2** was identified as rutin (quercetin-3-O-rutinoside). Signals in the proton spectrum were assigned as those of rutin assigned in the literature [19, 20], as well as a 1H-NMR and a HSQC spectrum of standard rutin recorded in this study.

Compound** 1** was identified as a glycoside flavonoid composed of the aglycone quercetin by comparison of the proton spectrum with that of rutin, with a triglycoside attached to the O-3 position according to MS and NMR data obtained. The sugar molecules were identified by GC-MS analysis of the hydrolysed sugars, showing that the triglycoside consisted of two sugar molecules of glucose and one of rhamnose. However, we were not able to identify the exact structure of the triglycoside.

**Figure 4:**
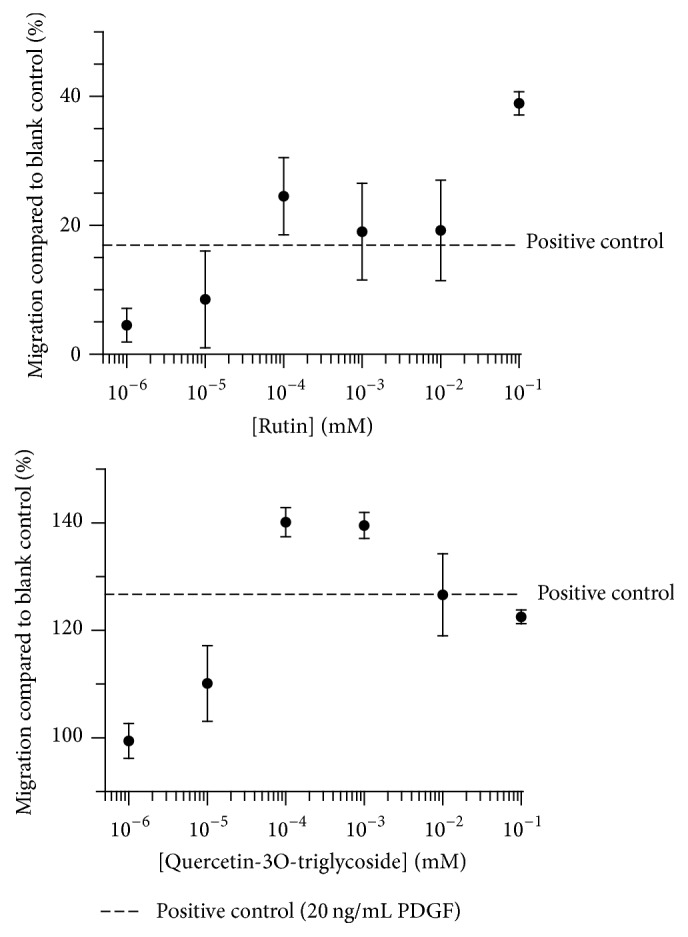
Concentration dependent migration of NIH 3T3 fibroblasts when incubated with rutin or the quercetin-3O-triglucoside from fraction 3.

Different concentrations of standard rutin and the isolated quercetin-triglycoside were tested in the wound healing scratch assay. The results showed a concentration dependent activity of both compounds (Figure 4), with a concentration of 10 nM giving results comparable to 20 ng/mL PDGF.

In previous studies rutin has shown wound healing activity in vitro [21] as well as in vivo [22]. Rutin has also shown antioxidant activity [23] as another approach for wound healing. The poor solubility of rutin in aqueous media has been overcome in a study where rutin is formulated as an injectable bioactive hydrogel of rutin-conjugated chitosan. This formulation contributed to improve the healing of dermal wounds [24]. Rutin has also been shown to reduce the healing time for injuries when taken orally in a clinical study [25]. Thus, rutin may hold some promise as an agent in wound healing.

## 4. Conclusion

*Allophylus spicatus, Philenoptera cyanescens, Melanthera scandens, Ocimum gratissimum,* and* Jasminum dichotomum *showed proliferation and/or migration of fibroblasts in the scratch assay. Thereby the historical use of these plants as wound healing remedies in Ghana is supported. The wound healing activity was attributed to the glycoside flavonoids rutin and a triglycoside of quercetin in* P. cyanescens*.

## Figures and Tables

**Table 1:** Plant species historically used for wound healing in Ghana.

Plant species	Family	Voucher	Collection site location	Plant part
*Aframomum melegueta *K. Schum.	Zingiberaceae	JS 224	N 05°51 17.0, W 00°10 30.2	Semen
*Allophylus spicatus *(Poir.) Radlk.	Sapindaceae	JS 206	N 05°39 25.3, W 00°11 08.2	Radix
				Herba
*Annona senegalensis *Pers.	Annonaceae	JS 253	N 05°39 24.6, W 00°11 29.5	Folium
*Cissus quadrangularis *L.	Vitaceae	JS 256	N 05°39 14.0, W 00°11 06.6	Herba
*Gymnanthemum coloratum (Willd.) H. Rob. & B. Kahn*	Asteraceae	JS 268	N 05°49 59.1, W 00°07 03.3	Folium cum Flos
				Radix
*Indigofera pulchra *Willd.	Fabaceae	JS 270	N 05°49 59.1, W 00°07 03.3	Herba
*Jasminum dichotomum *Vahl	Oleaceae	JS 273	N 05°54 21.1, W 00°00 18.5	Folium
*Leonotis nepetifolia var. africana *(P. Beauv.) J. K. Morton	Lamiaceae	JS 278	N 05°54 21.1, W 00°00 18.5	Herba
*Melanthera scandens *(Schum. & Thonn.) Roberty	Asteraceae	JS 220	N 05°51 49.8, W 00°10 01.0	Herba
*Millettia thonningii *(Schumacher) Baker	Fabaceae	JS 294	N 05°39 13.3, W 00°11 12.5	Cortex
*Ocimum gratissimum *L.	Lamiaceae	JS 223	N 05°51 49.8, W 00°10 01.0	Herba
*Philenoptera cyanescens *(Schum. & Thonn.) Roberty	Fabaceae	JS 204	N 05°42 59.4, W 00°10 35.5	Folium cum Fructus
*Rourea coccinea *(Schumach. & Thonn.) Benth.	Connaraceae	JS 248	N 05°39 24.1, W 00°11 11.0	Folium
				Radix
*Thonningia sanguinea *Vahl	Balanophoraceae	JS 296	N 05°51 10.6, W 00°10 41.9	Herba
*Trichilia monadelpha *(Thonn.) J. J. de Wilde	Meliaceae	JS 260	N 05°51 15.9, W 00°10 30.1	Cortex
*Triumfetta rhomboidea *Jacq.	Malvaceae	JS 272	N 05°54 52.3, W 00°02 17.0	Radix
*Uvaria ovata *(Vahl ex DC.) Hook. f. & Benth.	Annonaceae	JS 207	N 05°39 26.0, W 00°11 05.4	Folium
				Cortex
				Radix
				Folium cum Flos
